# Re-treatment With Adjuvant Radioactive Iodine Does Not Improve Recurrence-Free Survival of Patients With Differentiated Thyroid Cancer

**DOI:** 10.3389/fendo.2019.00671

**Published:** 2019-09-27

**Authors:** Clément Bouvet, Bertrand Barres, Fabrice Kwiatkowski, Marie Batisse-Lignier, Meryem Chafai El Alaoui, Philippe Kauffmann, Florent Cachin, Igor Tauveron, Antony Kelly, Salwan Maqdasy

**Affiliations:** ^1^Service de Médecine Nucléaire, CLCC Jean Perrin, Clermont-Ferrand, France; ^2^Université Clermont Auvergne, Faculté de Médecine, Clermont-Ferrand, France; ^3^Service de Biostatistique, CLCC Jean Perrin, Clermont-Ferrand, France; ^4^CHU Clermont-Ferrand, Service d'Endocrinologie, Diabétologie et Maladies Métaboliques, Clermont-Ferrand, France; ^5^Laboratoire GReD: UMR Université Clermont Auvergne-CNRS 6293, INSERM U1103, Aubière, France; ^6^Service de Chirurgie Thoracique, CLCC Jean Perrin, Clermont-Ferrand, France; ^7^UMR INSERM 1240 “Molecular Imaging and Theranostic Strategy”, Clermont Auvergne University, Clermont-Ferrand, France

**Keywords:** differentiated thyroid cancer, adjuvant RAI, recurrence, persistence, re-operation, re-treatment

## Abstract

**Introduction:** Loco regional persistence or recurrence of differentiated thyroid cancer (DTC) is frequent despite initial thyroidectomy and radioactive iodine therapy (RAI). The aim of this study was to analyze the impact of a complementary adjuvant RAI (Ad-RAI) on disease recurrence following re-operation on patients with locally persistent or recurrent DTC.

**Patients and Methods:** A retrospective study of 85 patients with DTC was conducted. All patients were initially treated with total thyroidectomy and RAI, and re-operated for a locally persistent or recurrent disease. Propensity score was calculated to predict the impact of Ad-RAI on survival after reoperation, and to reduce the bias of the limited sample size and the prognostic tests.

**Results:** 49 (58%) patients were re-treated with Ad-RAI after re-operation while 36 (42%) were only followed up. Disease recurrence after re-treatment (re-operation ± Ad-RAI) was detected in 31 patients (36.5%). In multivariate analysis, age >55 years (HR: 3.9 [1.6; 9.5]; *p* < 0.00001) was the main poor prognostic factor for recurrence-free survival. Three parameters independently influenced the decision to administer ad-RAI: low number of previous RAI administrations, Nx before re-operation, and pTg > 30 μg/l. These parameters were incorporated in the Propensity score calculation. If ad-RAI tended to improve recurrence-free survival (median survival 17.4 vs. 10.9 months), adjustment using the Propensity score removed any difference between the groups (*p* = 0.54), confirming the limited value of ad-RAI.

**Conclusion:** In patients with locally persistent or recurrent DTC, age is the main independent prognostic factor. Adjuvant RAI does not improve recurrence-free survival of DTC patients.

## Introduction

Differentiated thyroid cancer (DTC) is the most frequent endocrine cancer. Despite the increased incidence of DTC in recent decades, disease progression is usually slow and 10-year survival is excellent (1, 2). Nevertheless, post-surgical loco regional disease persistence and recurrence are detected in about 20 and 30% of patients, respectively (3–5). These two conditions are defined by the presence of morphological anomalies, detected by imaging techniques (e.g., ultrasound, CT scan, PET-CT), and/or biological anomalies (e.g., persistently elevated thyroglobulin levels >1 μg/l under suppressed TSH and/or >10 μg/l after TSH stimulation) (6).

The prognostic value of re-operation in patients with locally persistent or recurrent disease has been demonstrated previously (7). According to the American Thyroid Association (ATA), re-operation is recommended for persistent or recurrent loco regional disease when the lesion exceeds 8–10 mm on the smallest diameter (6). For smaller lesions, active follow up is suggested (8). Thus, these recent modifications in the management of DTC support a less aggressive strategy, limiting the indications of re-operation and re-treatment with adjuvant radioactive iodine ^131^I (RAI) for more extensive disease. In a retrospective study on 45 patients, Yim et al. demonstrated that adjuvant RAI did not reduce thyroglobulin levels in 23 patients with persistent or recurrent disease, including distant metastases (9). Hirsch et al. analyzed 44 patients who were re-operated before re-treatment. In this group, 47% had a persistent disease despite re-treatment with RAI (10). Whether a treatment with complementary or adjuvant RAI after re-operation is useful or not remains a matter of debate, as no randomized controlled clinical trials have been conducted.

This study aims to identify the prognostic factors, including adjuvant RAI, that influence recurrence-free survival in patients re-operated for persistent or recurrent DTC following initial ablative management by surgery and RAI.

## Patients and Methods

### Inclusion

**Figure 1:**
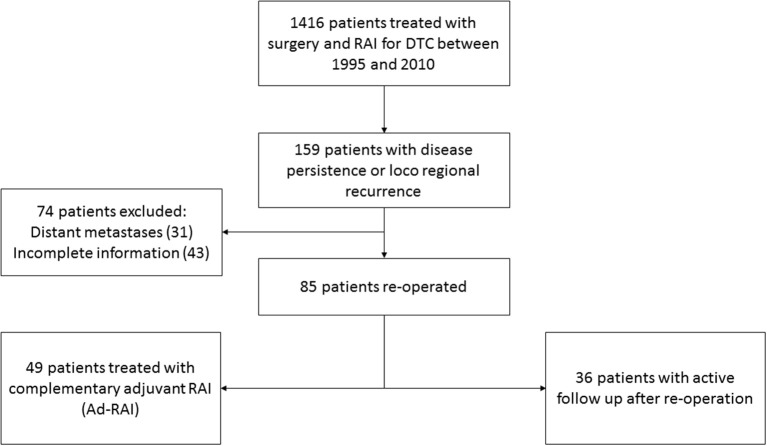
Flow chart of inclusion of patients in the study.

We have conducted a retrospective study in the Department of Nuclear Medicine at the Jean Perrin Comprehensive Cancer Center (Université Clermont Auvergne). Clinical and biological data obtained, as well as imaging results, are registered in our database. Of the 1,416 patients treated for differentiated thyroid cancer between 1995 and 2010, 159 (11.2%) patients presented a structurally persistent or recurrent disease. Management and clinical follow up of persistent and/or recurrent disease was done according to the French national guidelines for the management of differentiated thyroid cancer, and was systematically validated through weekly multidisciplinary meetings. Structural disease recurrence/persistence was confirmed through morphological evaluation (cervical ultrasound, CT-scan, ^18^FDG PET-CT) to identify proven malignant tissue in the cervical region. Due to incomplete follow up or to distant metastases, 74 patients were excluded. The remaining 85 patients with DTC who presented with loco regional (cervical) cancer persistence or recurrence after initial thyroidectomy and RAI for remnant ablation were included. These patients received second surgical intervention (re-operated) for disease persistence or recurrence. Among them, 49 patients had a complementary adjuvant RAI (Ad-RAI) performed shortly after re-operation, while 36 patients without an adjuvant RAI were only followed-up (Figure 1).

The impact of ad-RAI on recurrence-free survival was evaluated in comparison to patients without adjuvant RAI.

### Ethical Issues

Each patient was informed about this retrospective study before inclusion and analysis. No signed consent was necessary according to French legislation. Authorization and approval for data inclusion in this study was requested through individual letters sent to each patient describing the study's purpose and methods. Patients' opposition was respected, and their data was excluded from the database. Included patients had the right to obtain the main results of study if they wished. Study ethics approval was obtained on June 30, 2017 (CECIC Rhône-Alpes-Auvergne, Grenoble), IRB 5921.

### Thyroid Cancer Ablation Protocol and Follow-Up

All patients had undergone total thyroidectomy, either at our institution or at a regional hospital, followed by RAI ablation (100 mCi). A detailed histopathological description of the DTC with its particularities (cell types), extension, and staging was performed for all patients. RAI ablation was systematically decided through a multi-disciplinary meeting.

All RAIs were administered under thyroid hormone withdrawal (THW). THW consisted of LT4 withdrawal during the 5 weeks prior to RAI administration. LT4 was substituted with LT3 during the first 3 weeks, then stopped. Stimulated thyroglobulin (sTg) and anti-Tg antibodies were evaluated on the day of RAI therapy. A diagnostic whole body scintiscan (WBS) was performed on day 5 after RAI in order to document the RAI avidity of any structural disease or thyroid remnant. Six to twelve months after RAI administration, measurement of rhTSH-stimulated serum Tg, WBS, and cervical ultrasonography were performed to evaluate response to initial treatment and Tg status. Afterwards, patients were followed up through clinical checkup, laboratory testing and ultrasonography. In selected cases, additional CT imaging at varying intervals were performed.

TNM staging system of AJCC 2010 (7th edition) was used. Thyroid capsule invasion (i.e., the difference between T2 and T3) and tumor size were considered in our cohort in order to be compatible with the new TNM system published just after the completion of the collection of our variables (2017).

### Thyroglobulin Measurement

Thyroglobulin measurement methodology remained unaltered throughout the duration of the study. The measurement was performed in the laboratory of radiopharmacology in the Jean Perrin Center. Immuno-radiometric assay with coated tubes (CisBio:TgIRMA) was used for this purpose, with a nadir of 0.1 μg/l. Evaluation of possible antibody interference was thoroughly performed.

### Definitions

Initial management or treatment is defined as a primary thyroidectomy followed by remnant ablation by RAI. Long-term remission, deemed an excellent response, is defined by absence of biochemical and/or morphological signs of disease, and absence of any need for a second treatment (re-operation or ad-RAI).

Persistent disease is defined based on the identification of a structural and/or biochemical persistence of cancer after the initial management.

Recurrent disease is defined as evidence of disease re-emergence after an initial remission (i.e., structural lesion identified by US, WBS, and/or biochemical increment of thyroglobulin). Disease recurrence was detected as a result of regular radiological and biochemical monitoring of these patients.

### Evaluation of Long-Term Remission and Disease Recurrence/Persistence

Biological and morphological evaluation was performed 6–12 months after RAI. Patients were evaluated under rhTSH stimulation. Undetectable sTg, normal cervical ultrasound, and normal radio isotope scan (0.18 GBq of ^131^I) suggest a long-term remission or excellent response.

Any anomaly persisting during the initial evaluation (e.g., incomplete biochemical and/or structural response) is considered disease persistence. Disease recurrence is defined as the appearance of biochemical or structural anomalies in patients previously considered in remission.

### Statistical Analyses

The statistical analysis aimed to describe the general characteristics of the population at inclusion. Quantitative parameters are expressed as a mean with standard deviation (SD), or median [range] in cases of non-Gaussian distribution. Categorical parameters are expressed as population per category and as frequencies (%). Relationships between variables were analyzed with Chi^2^ for qualitative parameters, and Anova and Kruskal-Wallis H tests for quantitative parameters (depending on normality and/or homoscedasticity of distributions).

The prognostic value of different factors affecting recurrence-free survival was estimated using Kaplan-Meier curves. Statistical comparisons were achieved with Log-Rank and Mantel-Haenszel tests. The study of the impact of complementary RAI on survival without recurrence was adjusted on the Propensity score using the Cox model. Propensity score was incorporated in the statistical analyses to limit bias of the prognostic tests.

Propensity score was calculated based on significant parameters in univariate analysis (*p* ≤ 0.05). They were then included in a multiple logistic regression model. The resulting probability to be re-treated by RAI was used as the propensity score (11, 12). Score validity was verified by ROC analysis and an AUC > 0.7 was considered acceptable.

## Results

### Population Characteristics

Among the 85 patients included in the study, 59 were females (69.4%). Mean age at re-operation was 50.5 years (*SD*: 18.7). Papillary cancer was the predominant histological type of carcinoma in this cohort (92.9%). The median follow up period was 10.3 years [0.5–21.4]. The main characteristics of the population are summarized in Table 1.

**Table 1:** General characteristics of the patients included in the study.

**Parameter**	**Total (85 patients)**	**Follow up (36 patients)**	**Ad-RAI (49 patients)**	***P***
**Age at re-operation (years)**	50.5 (18.7)	57.7 (18.9)	44.7 (16.9)	0.002
**No. Female (%)**	59 (69.4%)	26 (72.2%)	33 (67.3%)	0.46
**Papillary cancer**	79 (92.9%)	34 (94.4%)	45 (91.8%)	0.99
**Initial stimulated pTg (μg/l)**	156 (362)	40 (56)	239 (456)	0.02
**T**				0.29
T1	13 (15.3%)	9 (25%)	4 (8.2%)	
T2	10 (11.7%)	5 (13.9%)	5 (10.2%)	
T3	37 (43.5%)	15 (41.7%)	22 (44.9%)	
T4	13 (15.3%)	4 (11.1%)	9 (18.3%)	
Tx	12 (14.1%)	3 (8.3%)	9 (18.4%)	
**N**				0.32
0	3 (3.5%)	3 (8.3%)	0	
≤ 5 LN	33 (38.8%)	9 (25%)	24 (49%)	
>5 LN	15 (17.7%)	3 (8.3%)	12 (24.5%)	
Nx	34 (40.0%)	21 (58.4%)	13 (26.5%)	
**Scintigraphy before re-operation**				0.002
CR	43 (50.6%)	15 (41.7%)	28 (57.1%)	
Lymph nodes	22 (25.9%)	6 (16.6%)	16 (32.7%)	
No uptake	20 (23.5%)	15 (41.7%)	5 (10.2%)	
**Stimulated Tg after reoperation (μg/l)**	21 (360)	15 (319)	52 (377)	0.44
**Disease evolution**				0.72
ER	56 (65.9%)	22 (61.1%)	34 (69.4%)	
BP	10 (11.8%)	5 (13.9%)	5 (10.2%)	
SP	19 (22.3%)	9 (25%)	10 (20.4%)	
**Disease recurrence (%)**	31 (36.5%)	14 (38.9%)	17 (34.7%)	0.22

*Quantitative parameters are expressed as mean with SD. Qualitative parameters are expressed as number (%). pTg is measured under thyroid hormone withdrawal (THW) during the ablative radioactive iodine therapy (RAI) prior to re-operation. Ad-RAI, adjuvant RAI; CR, Cervical residue; ER, Excellent response; BP, Biochemical persistence; SP, Structural persistence*.

### Factors Associated With Adjuvant RAI (Ad-RAI) Administration

We compared patients who received an ad-RAI after re-operation (Ad-RAI group) with those who had only active follow-up after re-operation (follow-up group). Ad-RAI group patients were younger (44 years old in the Ad-RAI group vs. 57 years old in the follow-up group, *p* < 0.002) (Table 1). Additionally, lymph node involvement was more frequent in the Ad-RAI group (73.5 vs. 33% of patients; *p* < 0.00001), pre-surgical whole body scintigraphy with a positive fixation (cervical or lymph node) was more frequent in the RAI-Ad group (89.8 vs. 58.3% of patients; *p* < 0.01), and pre-ablation thyroglobulin levels after THW were higher in the Ad-RAI group (239 vs. 40 μg/l; *p* < 0.05) (Table 1).

### Recurrence-Free Survival of the Cohort

**Figure 2:**
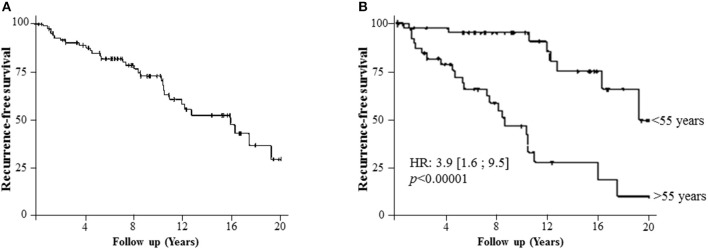
The recurrence-free survival of the cohort. **(A)** Recurrence-free survival (median 15.9 years) of the cohort demonstrating a steady decline of survival with time. **(B)** Recurrence-free survival in patients younger or older than 55 years old.

Disease recurrence after re-treatment was detected in 31 patients (36.5%), with a median recurrence-free survival of 15.9 years. The survival curve showed a steady decline in recurrence-free survival with time (Figure 2A). The frequency of disease recurrence was stable for years following re-operation. The annual incidence of disease recurrence was 3.5%.

After a multivariate COX analysis, the main poor prognostic factor in terms of recurrence-free survival was age over 55 years (HR: 3.9 [1.6; 9.5]; *p* < 0.00001) (Figure 2B). Indeed, patients older than 55 years have a higher risk of recurrence independently of time, employment or not of ad-RAI. Sex, initial LN invasion status (Nx or N1), and initial radio-isotope scintigraphy results had no influence on disease recurrence.

### Influence of Ad-RAI on Remission and Recurrence-Free Survival

After reoperation, thyroglobulin levels were statistically indifferent between both groups (52 vs. 15 μg/l (*p* = 0.44) (Table 1). Disease remission rates in the Ad-RAI group and the follow-up group did not differ (61 vs. 69%). Recurrence rates were statistically indifferent (39 vs. 34%) (Table 1). Follicular cancer was described in 6 patients (2 in the follow up group and 4 in the Ad-RAI group). All of them suffered from disease persistence or recurrence with or without ad-RAI.

**Figure 3:**
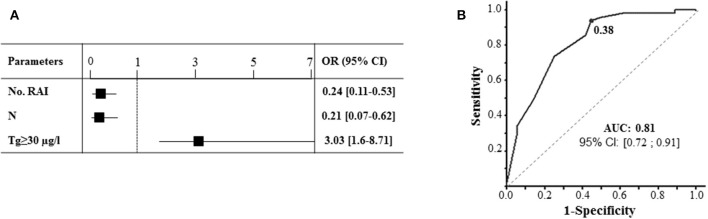
Estimation of Propensity score necessary for adjustment of the effect of adjuvant RAI on disease recurrence. **(A)** Multivariate analysis identifying parameters independently influencing the decision of administration of ad-RAI. These parameters are incorporated in the estimation of propensity score calculation. “No. RAI” represents the number of RAI therapies administered before re-operation; N represents the initial lymph node invasion status; pTg is measured under THW stimulation during the radioactive iodine therapy prior to re-operation. **(B)** ROC analysis with estimation of AUC to validate the Propensity score.

**Figure 4:**
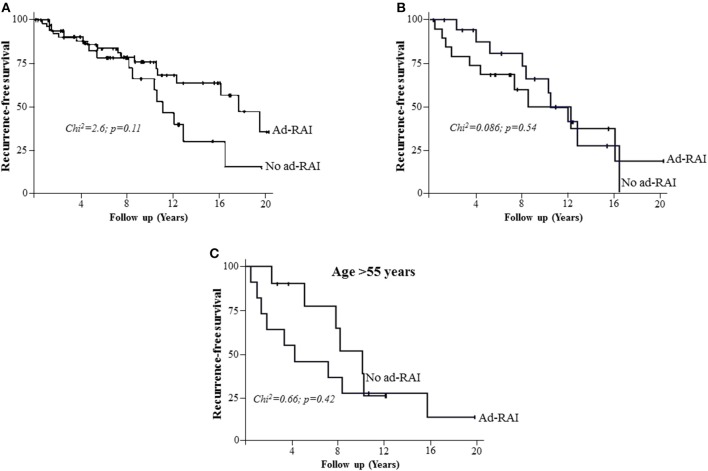
The impact of adjuvant RAI on the recurrence-free survival. **(A)** Kaplan Meier analysis demonstrating the recurrence-free survival in patients with (Ad-RAI) or without adjuvant RAI (No Ad-RAI). No significant difference between the populations was identified. **(B)** Kaplan Meier analysis, adjusted using the Propensity score, demonstrating the recurrence-free survival in patients with (Ad-RAI) or without (No Ad-RAI) adjuvant RAI. **(C)** Kaplan Meier analysis demonstrating the recurrence-free survival in patients older than 55 years old with (Ad-RAI) or without (No Ad-RAI) adjuvant RAI.

Three variables were considered significant and independently predicted the choice of an ad-RAI after re-operation. These variables were used in the calculation of the Propensity score, and included: low number of previous RAI treatments, presence or absence of initial LN involvement (Nx or N1; *p* < 0.00001) and pTg ≥ 30 μg/l (Figure 3A). ROC analysis validated the resulting Propensity score, with an AUC > 80 (Figure 3B). Without any adjustment, the recurrence-free survival of two groups of patients (with or without adjuvant RAI) were not significantly different (*p* = 0.11), but tended to be in favor of the ad-RAI (median 17.4 vs. 10.9 months) (Figure 4A). The multivariate analyses adjusted on the Propensity score eliminated any difference between both groups of patients (*p* = 0.54), as demonstrated by Kaplan Meier curves (Figure 4B). Furthermore, multivariate analyses and Kaplan Meier curves did not show any significant benefit of ad-RAI in patients with a poor prognostic factor (age >55 years) (Figure 4C).

## Discussion

In this retrospective study, we analyzed the general prognosis of patients with DTC who were re-treated for recurrent and/or persistent loco regional (cervical) disease despite an initial thyroidectomy and RAI remnant ablation. Age, the main prognostic factor influencing recurrence-free survival, and the effect of re-treatment by ad-RAI were evaluated and adjusted using Propensity score. This study demonstrates that ad-RAI does not impact recurrence-free survival of DTC patients.

Disease persistence occurred in 34% of patients, while disease recurrence occurred in 36.5% of the patients over 15 years. Similarly, many other retrospective studies estimated the risk of disease recurrence between 1 and 50% (13–19). In addition, the survival curve of the whole population demonstrated a steady decline in the recurrence-free survival. This evidence confirms that patients with a loco regional recurrence or biochemical persistence should be followed up on a long term basis and may be considered a high risk group for disease recurrence. It is believed that this variability in the recurrence rate is highly dependent on the risk rate of DTC and the response to the initial management (6). Indeed, ATA recommendations suggest that the clinical outcome of the “*structural incomplete response category”* is usually disease persistence or recurrence in about 50–85% of the patients despite further therapies (7, 20–22). Such patients have worse clinical outcomes than the biochemical persistence category of patients (20).

Age constitutes a major prognostic element, and consequently, it is incorporated in the staging system (23, 24). Age over 55 years was the main poor prognostic factor in our cohort. Although survival rates in patients over the age of 45 are reduced (6, 25, 26), patients older than 55 years are known to have a higher risk of recurrence and lowest survival rates. Consequently the new staging AJCC/TNM system modified the age cut-off to 55 years (23, 24). Other factors like histological subtypes or the presence of Hashimoto's thyroiditis could change the prognosis of the patients either by increasing or reducing the recurrence rates (27–30).

Compared to the 36 patients who were only followed up after re-operation, 49 patients were re-treated with ad-RAI. After a median follow up of 10 years, no significant difference, in terms of recurrence-free survival, was noticed between patients with or without adjuvant RAI. Indeed, few studies have evaluated the impact of re-treatment with RAI on the prognosis of patients with a persistent or recurrent loco regional DTC. Yim et al. retrospectively studied 45 patients with high Tg levels who received an ad-RAI treatment (23 patients) or follow-up only (22 patients) after re-operation, and demonstrated no benefit of Ad-RAI treatment in terms of reduction of survival without recurrence or Tg levels (9). In this study, 8 patients in the ad-RAI group and 5 patients in the follow up group had disease recurrence (29% of patients). Hirsch et al. analyzed 114 patients with a persistent structural or biochemical disease after initial treatment who obtained a second dose of RAI. Only 44 patients had complete data and were re-operated on before re-treatment. In this group, 47% had a persistent disease despite the retreatment (10). In this study, no control group exists. Other studies have demonstrated a spontaneous reduction in thyroglobulin levels without further RAI in patients who previously received high doses of RAI (31, 32). With these results, ad-RAI seems hazardous for such patients and re-operation for larger tumors seems sufficient to improve the survival in patients with loco regional recurrence or persistence (7, 22). However, for smaller tumors, early re-operation could increase the risk of incomplete surgery and persistent disease. Active follow up could be useful in such patients in order to better localize the tumor burden to optimize the surgical management.

The limitations of this study include the limited sample size and the non-randomization. Furthermore, selection bias could exist due to the retrospective nature of the inclusion, as patients with Ad-RAI had higher disease burden (Tg levels, lymph node involvement, scintigraphic data before reoperation). Despite the initial difference between both groups, thyroglobulin levels after reoperation were not different. These results reflect the impact of reoperation on disease burden especially in patients with high initial thyroglobulin levels (ad-RAI group) (7). Thus, the prognosis of patients with higher disease burden in the ad-RAI group could be considered as subjectively improved as their remission and recurrence rates were aligned to the follow up group. However, this benefit is largely contributed to reoperation rather than re-treatment with RAI.

Besides, there is no randomized controlled trial evaluating the benefits of ad-RAI. Unfortunately, Yim et al.'s study which seeks to address this issue has limitations related to the general guidelines in the management of DTC. Indeed, it will be difficult to design a randomized prospective study on patients with a high disease burden with “simple follow up” vs. Ad-RAI.

However, our data are regularly and prospectively registered in our database. In order to overcome these limitations, we used a Propensity score as previously described (11, 12) and this is the best methodology when no prospective randomized trial can be performed.

## Conclusion

Adjuvant RAI in patients re-treated for locally persistent or recurrent DTC did not improve the recurrence-free survival of patients. The recurrence rate was constant with time and was mainly dependent on age. Re-operation for larger tumors appears sufficient to improve survival in patients with loco regional disease recurrence or persistence.

## Data Availability Statement

All datasets generated for this study are included in the manuscript/supplementary files.

## Ethics Statement

All procedures performed in studies involving human participants were in accordance with the ethical standards of the institutional and/or national research committee and with the 1964 Helsinki declaration and its later amendments or comparable ethical standards. All individual participants included in the study were informed and their opposition was respected. Study ethics approval was obtained on June 30, 2017 (CECIC Rhône-Alpes-Auvergne, Grenoble), IRB 5921.

## Author Contributions

CB, BB, and AK collected data. FK completed statistical analyses. PK, MB-L, IT, AK, FC, MC, and SM participated in the management of patients. AK, FC, IT, MB-L, and SM designed the study, carefully reviewed the results, and reviewed the paper. SM wrote the paper.

### Conflict of Interest

The authors declare that the research was conducted in the absence of any commercial or financial relationships that could be construed as a potential conflict of interest.
